# GABA_B_R/GSK‐3*β*/NF‐*κ*B signaling pathway regulates the proliferation of colorectal cancer cells

**DOI:** 10.1002/cam4.686

**Published:** 2016-04-05

**Authors:** Qing Shu, Jun Liu, Xiupeng Liu, Sufang Zhao, Hualin Li, Yonggang Tan, Jianming Xu

**Affiliations:** ^1^ Department of gastroenterologyThe Affiliated Clinical College ShenZhen Second People HospitalShenzhenChina; ^2^ Anhui Medical UniversityHefeiChina

**Keywords:** Cell cycle, colorectal cancer, GABA_B_R, GSK‐3*β*, NF‐*κ*B, proliferation

## Abstract

Colorectal cancer is one of the leading causes of highly fatal cancer‐related deaths in the whole world. Fast growth is critical characteristic of colorectal cancer, the underlying regulatory mechanism of colorectal cell fast proliferation remains largely unknown. Here, we reported that activation of metabotropic *γ*‐Aminobutyric acid receptor (GABA_B_R) signaling significantly inhibited the colorectal cell HT29 proliferation by arresting the cell at G1 phase. Inhibition of GABA_B_R activated GSK‐3*β* by reducing the phosphorylation level of GSK‐3*β*. Activation of GSK‐3*β* blocked the function of GABA_B_R signaling on repressing cell proliferation. We further found that GABA_B_R activation inhibited NF‐*κ*B activity. The promotion of cell proliferation caused by downregulation of GABR_B_R could be blocked by inhibition of NF‐*κ*B activation. Overall, activation of GABA_B_R leaded to inhibition of GSK‐3*β* activation to repress the NF‐*κ*B function during colorectal cancer cell proliferation. This study revealed critical function of GABA_B_R/GSK‐3*β*/NF‐*κ*B signaling pathway on regulating proliferation of colorectal cancer cell, which might provide a potential therapeutic target for clinical colorectal cancer treatment.

## Introduction

Colorectal cancer (CRC) is a major cause of cancer mortality worldwide with approximately 694,000 recorded deaths from the disease in 2012 1, 2. It is estimated that more than 1 million cases are diagnosed annually and around 600,000 of them are death 3. CRC is an ideal model of research of the molecular pathogenesis of cancer, due to the ease of obtaining biopsy material and the understanding of the development of invasive carcinoma, from normal epithelium to polyps, and carcinoma 4. Colorectal carcinogenesis is a multistep process that requires the accumulation of genetic/epigenetic aberrations in signal transduction pathways 5. However, the regulation of the colorectal cancer cell proliferation process is limited and needs further investigation.

*γ*‐aminobutyric acid (GABA), the main inhibitory neurotransmitter in the vertebrate brain, acts on ionotropic (GABA_A_ or GABA_C_) and metabotropic (GABA_B_) receptors, is an inhibitory neurotransmitter 2. An increasing number of studies have demonstrated the potential roles of neurotransmitter receptors in tumors like GABA_B_ receptors, as a key therapeutic targets in neurological diseases, also can suppress the proliferation of various human tumor cells 2. Wang and his partners have reported that the proliferation of hepatocarcinoma cells Bel‐7402 and Huh‐7 was inhibited by baclofen, an activator of GABA_B_R, in a dose‐dependent manner. Systemic administration of baclofen significantly suppressed the growth of Bel‐7402 xenograft induced in nude mice 6. In addition, Opolski's laboratory found that a remarkable growth inhibition of experimental mammary cancer 16/C was observed in mice treated with baclofen 7. However, the study about the regulation of proliferation and cell cycle in colorectal cancer cell is unknown and still need our further investigation.

Glycogen synthase kinase‐3*β* (GSK‐3*β*), a serine/threonine protein kinase, has been regarded as a potential therapeutic target for multiple human cancers 8. Growing evidences showed that GSK‐3*β* plays an important role in diverse cellular processes including proliferation, differentiation, motility, and survival 9. Recent report had been reported that it can regulate the proliferation of human ovarian cancer cells in vitro (SKOV3 and ES‐2 cells) as well as in vivo 10. Study also showed that the relevance of GSK‐3*β* as a target for controlling cell cycle progression and proliferative capacity in MCF7, highlighted the cotreatment of breast cancer 11. However, the deep molecular mechanism of the regulation by GSK‐3*β* on CRC and its signaling pathway worths further investigation.

Lately many reports show that nuclear factor‐*κ*B (NF‐*κ*B) not only plays an important role in the coordination of innate and adaptive immune responses and cell‐cycle regulation, but also proved to have a pivotal role in tumorigenesis 12. NF‐*κ*B is a family of five master transcription factors, including NF‐*κ*B1/p105, NF‐*κ*B2/p100, RelA/p65, RelB, and c‐Rel, which can form various heterodimers or homodimers and bind to consensus DNA sequences at promoter regions of responsive genes 13. NF‐*κ*B is activated in response to various stimuli (cytokines, growth factors, oncoproteins, stress signals) and can follow two distinct pathways: the canonical pathway and the noncanonical pathway 13, 14. Huili Li and his laboratory had demonstrated that inhibition of GSK‐3*β* could suppress the proliferation of colorectal cells by the downregulation of activity of NF‐*κ*B and NF‐*κ*B‐mediated target genes transcription, which may be of benefit for clinical outcome in patients suffering from colon cancer in future 8. Understanding of how GABA_B_R, GSK‐3*β*, and NF‐*κ*B participate in the CRC cell proliferation and cell cycles will be helpful in finding deep regulation mechanism of cancer cell in order to facilitate clinical application.

Here, we demonstrated that GABA_B_R played an important role in regulating the CRC cell proliferation. Activation of GABA_B_R leaded to inhibition of GSK‐3*β*. Repression of GSK‐3*β* lead to upregulation of cell proliferation could be rescued by inhibition of GABA_B_R. Further, inhibition of GSK‐3*β* could inhibit NF‐*κ*B so that to suppress the cancer cell proliferation. The downregulation of cell proliferation caused by inhibition of GABA_B_R could be rescued by repression of NF‐*κ*B. These results indicated that the critical function of GABA_B_R/GSK‐3*β*/NF‐*κ*B signaling in colorectal cancer cell proliferation, and might provide more insight into the specific roles of GABA_B_R to lay the foundation for further clinical application.

## Material and Methods

### Cell culture

Cells were cultured at 37°C, 5% CO_2_ in RPMI‐1640 medium (Gibco, New York, USA) with 10% fetal bovine serum (FBS) (Gibco), 100 U/mL of penicillin sodium (Invitrogen, Life Technologies, Carlsbad, California, USA), and 100 mg/mL of streptomycin sulfate (Invitrogen, Life Technologies).

GABAB R1 knockdown: The Plko.1 vector was used to construct shRNA vectors. The shRNA sequences were as follows: shRNA‐GABABR1‐1: 5′‐CCCGAATCTGCTCCAAGTCTTA‐3′; shRNA‐GABABR1‐2: 5′‐ ACCAAGCCACCAAGTACCTATA‐3′.

### Proliferation assay

Cells were seeded in the 96‐well plate for the MTS proliferation assay. Cell Titer 96^®^ AQueous One Solution Cell Proliferation Assay kit (Promega, Madison, Wisconsin, USA) was used to perform the MTS proliferation assay following the instruction. MTS is 3‐(4,5‐dimethylthiazol‐2‐yl) ‐5‐(3‐carboxymethoxyphenyl)‐2‐(4‐sul‐fophenyl)‐2H‐tetrazolium. The MTS tetrazolium compound can be bioreduced to be the colored formazan product that is solute in culture medium. The record absorbance at 490 nm was detected by microplate reader.

### Bromodeoxyuridine (BrdU) assay

A BrdU Cell Proliferation Assay kit (cat. no. 2752; EMD Millipore, Bedford, MA) was used to detect cell proliferation ability. Cells were seeded in 96 wells (2 × 103 cells/well) in the medium with 10 *μ*L BrdU solution. After incubation, phosphate buffer saline (PBS) solution with 4% paraformaldehyde was used to fix the cells for 15 min. Then, the cells were washed with PBS and treated with DNase for 15 min. The cells were washed with PBS. Then, BrdU antibody(Abcam, Cambridge, Massachusetts, USA)was added and the cells were incubated at 4°C for 8 h. Then, the cells were incubated with secondary antibody at room temperature for 60 min. Cell nucleus were dyed with 4′,6‐diamidino‐2‐phenylindole (DAPI). Then, the BrdU‐positive cells were counted.

### FACS assay

Single cell suspension was collected by digesting with trypsin and fixed in 70% ethanol for 12 h at 4°C treatment for fluorescence‐activated cell sorting (FACS) analysis. Then, cells were washed with PBS solution. Then, RNase A was added to the cells for 10 min. Propidium iodide (PI) was added to a final cell suspension with the concentration of 50 *μ*g/mL to stain the chromatin. Cells were analyzed using the flow cytometer.

### Quantitative real‐time PCR (qRT‐PCR)

The total RNA was isolated using RNAiso (Takara, Dalian, China). cDNA was subsequently reverse‐transcribed from mRNA by M‐MLV Reverse Transcriptase (Takara). The PCR included 40 cycles of amplification using the Stratagene Mx3000P system with SYBR Green qPCR Mix (BioRad, Hercules, California). Expression of target genes (2−^ΔΔCt^) was normalized against GAPDH. The sequence of primer used in the qRT‐PCR:

Cyclin D1PF 5′‐ CAATGACCCCGCACGATTTC‐3′.

Cyclin D1 PR 5′‐ CATGGAGGGCGGATTGGAA‐3′.

Rb1 PF 5′‐TTGGATCACAGCGATACAAACTT ‐3′.

Rb1 PR 5′‐ AGCGCACGCCAATAAAGACAT ‐3′.

RBL1 PF 5′‐ ACCACCAAAGTTACCACGAAG ‐3′.

RBL1 PR 5′‐ CCCCAATCATCCGAAAATTACCC ‐3′.

P21 PF 5′‐ TGTCCGTCAGAACCCATGC ‐3′.

P21 PR 5′‐ TGTCCGTCAGAACCCATGC ‐3′.

GAPDH PF: 5′‐ TGTGGGCATCAATGGATTTGG‐3′.

GAPDH PR 5′‐ ACACCATGTATTCCGGGTCAAT‐3′.

### Western blotting

SDS lysis buffer (Beyotime, Beijing, China) was used to lyse the cells for protein electrophoresis. Polyvinylidene fluoride (PVDF) membranes were used to transfer the protein and incubate the antibody. The primary antibodies are as followed: p‐GSK‐3*β* (Signaling way antibody, USA), GSK‐3*β* (Abcam), p‐IKB*α* (Abcam), IKB*α* (Abcam), p‐NF‐*κ*b‐p65 (Abcam), NF‐*κ*b‐p65(Abcam), GAPDH (Santa Cruz, Dallas, Texas, USA) diluted phosphate buffer saline (PBS) with 10% donkey serum albumin. The visualized signaling was performed by enhanced chemiluminescence (ECL)western blotting substrate kit (Thermo, Waltham, Massachusetts, USA).

### Statistical analyses

Student's *t*‐test was used to determined statistical significance. Values were presented as the mean ± SD. *means *P* < 0.05, **means *P* < 0.01, ***means *P* < 0.001, respectively.

## Results

### Activation of GABA_B_R inhibited the proliferation of colorectal cancer cell

**Figure 1:**
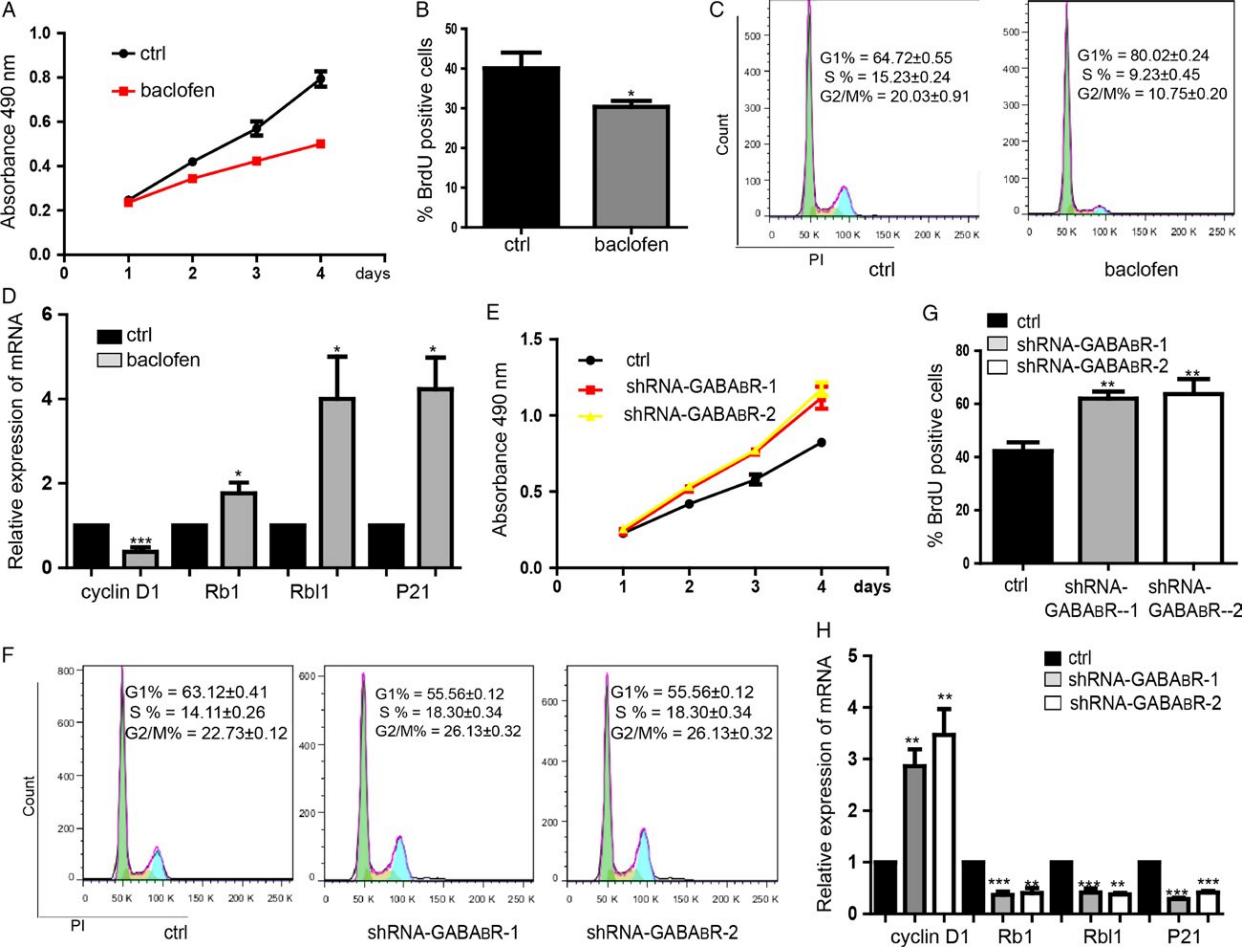
Activation of *γ*‐Aminobutyric acid receptor (GABA_B_R) inhibited the proliferation of colorectal cancer cell line HT29. (A) Baclofen inhibits HT29 proliferation detected by MTS proliferation assay. Ctrl means the cells treated with physiological saline. Data shown are means ± SD (*n* = 3). (B) BrdU incorporation assay showed the inhibition of proliferation caused by baclofen. Data shown are means ± SD (*n* = 4). *P < 0.05 versus the corresponding control. (C) Flow cytometry assay showed the cell cycle arrest at G1 phase by baclofen. Data shown are means ± SD (*n* = 5). (D) Expression level of mRNA of cell cycle‐related genes. Data shown are means ± SD (*n* = 4). *P < 0.05, ***P < 0.001 versus the corresponding control. (E) Detection of the proliferation of cells with downregualtion of GABA_B_R. Data shown are means ± SD (*n* = 5). (F) BrdU incorporation assay of cells with downregualtion of assay GABA_B_R. Data shown are means ± SD (*n* = 5). **P < 0.01 versus the corresponding control. (G) Cell cycle analysis by flow cytometry assay. Data shown are means ± SD (*n* = 5). (H) Expression level of mRNA of cell cycle‐related genes. Data shown are means ± SD (*n* = 4). **P < 0.01, ***P < 0.001 versus the corresponding control.

In order to determine the GABA_B_R signaling on regulating the colorectal cancer cell, we performed MTS assay on cell line HT29 and HCT116 to find that the capacity of colorectal cancer cells proliferation was repressed by baclofen (agonist of GABABR, 30 *μ*mol/L^−1^) (Figs. 1A and S1A). BrdU incorporation assay also showed that the cell proliferation of baclofen group was significantly inhibited (Figs. 1B and S1B). We then used the HT29 to investigate if activation of GABA_B_R can regulate cell cycle by flow cytometry and found that there was a significant increase in the proportion of cells in G1 phase, and a reduction in the proportion of cells in S, and G2/M phases (Fig. 1C). Meanwhile, we detected some cell cycle relative gene expression level by qRT‐PCR. We could find that the G1 phase‐related gene cyclin D1 was downregulated, while the cell cycle inhibitors Rb1, Rbl1, and P21 15 were upregulated (Fig. 1D). In contrast, knockdown of the GABA_B_R significantly promoted the capacity of proliferation (Figs. 1E and F, S1C, S1D). Additionally, the proportion of cells in G1 phase was decreased and increased in the S, G2/M phase by knockdown of the GABA_B_R (Fig. 1G). We also found that cyclin D1 was upregulated and the Rb1,Rbl1, P21 were downregulated(Fig. 1H).

### GABA_B_R signaling repressed colorectal cancer cell proliferation by inhibiting activity of GSK‐3*β*

**Figure 2:**
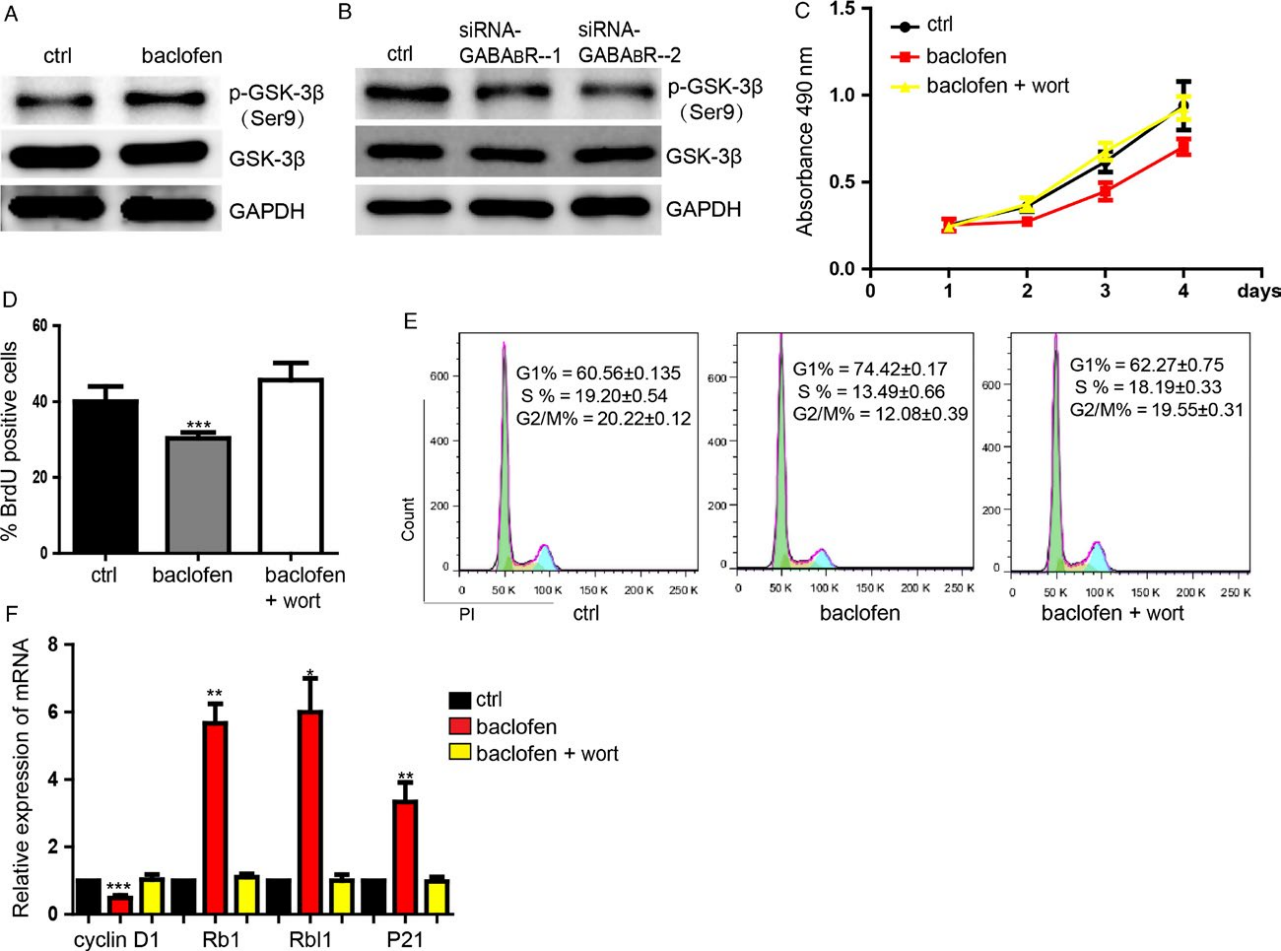
*γ*‐Aminobutyric acid receptor (GABA_B_R) signaling repressed colorectal cell proliferation by inhibiting activity of GSK‐3*β*. (A) Representative picture showed the increasing level of phosphorylation level of GSK‐3*β* caused by activation of GABA_B_R detected by western blot. (B) Downregulation of GABA_B_R activates the GSK‐3*β*. (C) MTS proliferation assay showed the function of activation of GABA_B_R can be blocked by activation of GSK‐3*β* by wort. Data shown are means ± SD (*n* = 4). (D) BrdU incorporation assay of the rescue experiment. Data shown are means ± SD (*n* = 4). ***P < 0.001 versus the corresponding control. (E) Flow cytometry assay. Data shown are means ± SD (*n* = 6). (F) The expression level of cell cycle‐related genes detected by qRT‐PCR. Data shown are means ± SD (*n* = 4). *P < 0.05, **P < 0.01, ***P < 0.001 versus the corresponding control.

To investigate whether GABA_B_R can regulate GSK‐3*β* to inhibit colorectal cancer cell proliferation and cell cycle, we performed further experiments. Western blot assay showed that activation of GABAR significantly increased the phosphorylation level of GSK‐3*β*, but could not regulate the expression of GSK‐3*β* (Fig. 2A). Downregulation of GABA_B_R inhibited the phosphorylation of GSK‐3*β*, which means the activation of GSK‐3*β* was increased. (Fig. 2B). In the contrary, knockdown of GABA_B_R could restrain the phosphorylation level of GSK‐3*β* to promoted GSK‐3*β* activation (Fig. 2B). Then, we found that wort (wortamannin, 10 *μ*mol/L^−1^), an agonist of GSK‐3*β*, could significantly block the proliferation repression caused by GABA_B_R signaling activation (Figs. 2C and D, S2A and B). Activation of GSK‐3*β* blocked the G1 phase arrest and the influence on cell cycle‐related genes caused by baclofen (Fig. 2E and F).

### GSK‐3*β*/NF‐*κ*B signaling regulates the colorectal cancer cell proliferation

**Figure 3:**
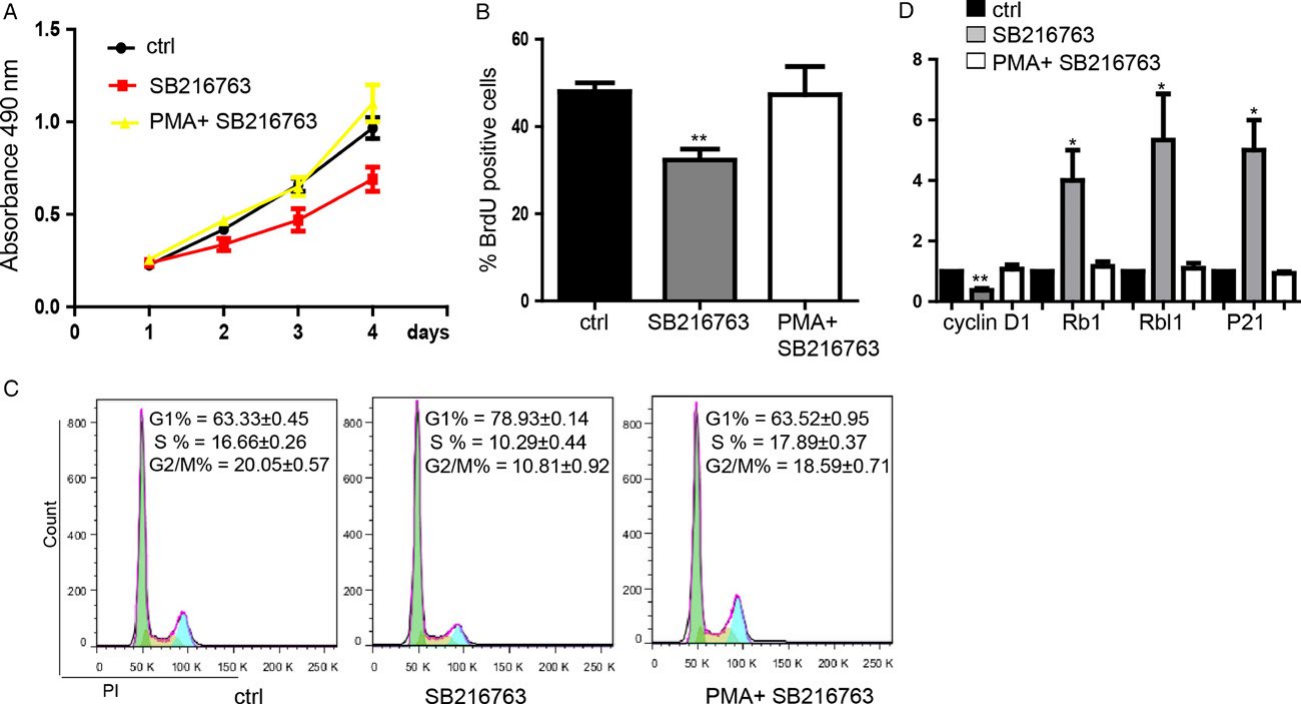
GSK‐3*β*/NF‐*κ*B signaling regulates the colorectal cancer cell proliferation. (A) MTS proliferation assay showed that the inhibition of cells proliferation caused by SB216763 could be rescued by PMA meanwhile. Data shown are means ± SD (*n* = 5). (B) BrdU incorporation assay for proliferation analysis. Data shown are means ± SD (*n* = 5). **P < 0.01 versus the corresponding control. (C) Flow cytometry assay showed that PMA blocked the influence of cell cycle caused by SB216763. Data shown are means ± SD (*n* = 3). (D) PMA blocked the influence of expression of cell cycle‐related genes caused by SB216763. Data shown are means ± SD (*n* = 4). *P < 0.05, **P < 0.01 versus the corresponding control.

In order to detect the regulatory mechanism of GSK‐3*β* on the proliferation, we further found that the repression of cell proliferation caused by inhibition of GSK‐3*β* by SB216763 could be blocked by Phorbol‐12‐myristate‐13‐acetate (PMA) (200 nmol/L^−1^) which is the agonist of NF‐*κ*B (Fig. 3A and B, S3A and B). Meanwhile, we found that PMA also could rescue the cell cycle which was arrested at G1 phase caused by SB216763 to be similar with control group (Fig. 3C). Besides, the influence of expression level of cyclin D1 and Rb1, Rbl1, P21 by SB216763 were also restored by PMA to be the similar level of control group (Fig. 3D). These results determined that suppression of GSK‐3*β*, which lead to restrain colorectal cancer cell proliferation, can be rescued by activation of NF‐*κ*B.

### GABA_B_R/NF‐*κ*B signaling pathway regulates the colorectal cancer cell proliferation

**Figure 4:**
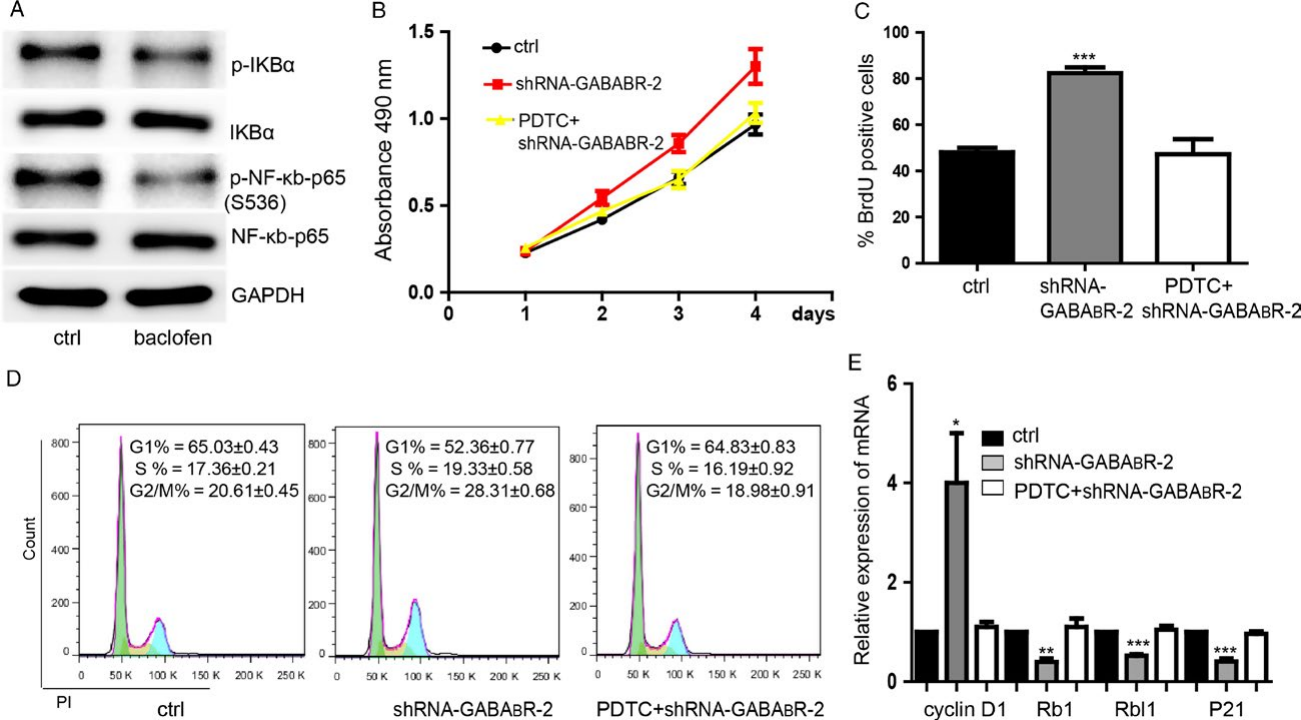
GABA_B_R/NF‐*κ*B signaling pathway regulates the proliferation. (A) Representative picture showed the detection of the influence of baclofen on NF‐*κ*B signaling. (B) MTS proliferation assay showed that the promotion of cells proliferation caused by downregulation of GABA_B_R could be rescued by PDTC meanwhile. Data shown are means ± SD (*n* = 3). (C) PDTC blocked the promotion of proliferation caused by downregulation of GABA_B_R. Data shown are means ± SD (*n* = 3). ***P < 0. 001 versus the corresponding control. (D) PDTC restored the cell cycle influence caused by downregulation of GABA_B_R. Data shown are means ± SD (*n* = 3). (E) PDTC restored the expression of cell cycle‐related genes influenced by GABA_B_R downregulation. Data shown are means ± SD (*n* = 3). *P < 0.05, **P < 0.01, ***P < 0.001 versus the corresponding control.

To determine whether GABA_B_R can inhibit NF‐*κ*B to repress the colorectal cancer cell proliferation, we first performed western blot experiment to find that phosphorylation level of p‐IKB*α* and p‐NF‐*κ*b‐p65 were downregulated compared with GAPDH,while the whole expression of IKB*α* and NF‐*κ*b‐p65 were not changed significantly, which mean that the NF‐*κ*B was activated (Fig. 4A). Then, we found that PDTC (100 *μ*mol/L^−1^), an inhibitor of NF‐*κ*B, could rescue the promotion of cell proliferation caused by downregulation of GABA_B_R (Fig. 4B and C, S4A and B). Inhibition of NF‐*κ*B restored the influence on cell cycle arrest at G1 phase caused by downregulation of GABA_B_R (Fig. 4D). Additionally, the influence on expression level of cyclin D1, Rb1, Rbl1, and P21 caused by downregulation of GABABR were also restored by inhibition of NF‐*κ*B (Fig. 4E). Additionally, in order to further detect how GABABR regulates the GSK‐3*β*/NF‐*κ*B signaling pathway in colon cancer cells, we performed the rescue experiment and found that inhibition of the inactivation of Akt kinase activity by MK‐2206 2HCl (15 *μ*mol/L^−1^) blocked the repression of proliferation caused by baclofen (Fig. S4C and D). MK‐2206 2HCl also restored the cell proliferation which was repressed by the GSK‐3*β* signaling inhibitor SB216763 (Fig. S4E and F). So, we hypothesized that Akt might be the mediator of the function of GABABR on regulating GSK‐3*β*/NF‐*κ*B signaling.

## Discussion

In summary, we uncovered that the GABA_B_R/GSK‐3*β*/NF‐*κ*B signaling pathway can regulate the proliferation of colorectal cancer. Upregulation of GABA_B_R significantly inhibited the colorectal cell HT29 proliferation. Further, inhibition of GSK‐3*β* activation could repress the cell proliferation which can be blocked by downregulation of GABA_B_R. Besides, we further confirmed that the promotion of cell proliferation caused by downregulation of GABR_B_R could be blocked by inhibition of NF‐*κ*B activation. These results might provide more insight into the specific roles of GABA_B_R/GSK‐3*β*/NF‐*κ*B signaling pathway in colorectal cancer cell and help us lay the foundation for further clinical application of CRC.

A metabotropic GABA_B_ receptor (GABA_B_R) is one of the GABA family members, which was originally identified as a major inhibitory neurotransmitter in the adult mammalian brain 16. Outside the brain, GABA and its receptors have been found in nonneuronal peripheral tissues, such as the gastrointestinal system 17, lung 18, and liver 19, including the biliary tract system 20. Studies have implicated GABA and its receptors as important players not only in synaptic inhibition, convulsion, pain, depression, and cognition 21, but also in the inhibition of cancer growth and tumor cell migration in, for example, colorectal carcinoma 22, breast cancer 23, and cholangiocarcinoma 16. For example, Zhang D and his lab members found that both the GABA synthetic enzyme (GAD65/67) and GABA_B_ receptor are expressed in mouse and human breast cancer cells. MCF‐7 human breast cancer cells and human breast cancer tissue. Baclofen, a GABA_B_R agonist, significantly promoted 4T1 cells invasion and migration in vitro and metastasis in vivo, an event that was attenuated by GABA_B_R antagonist CGP55845 24. Nevertheless, the mechanisms of GABA_B_R effect were not fully understood. In our study, we found that when GABA_B_R was upregulated by baclofen, the colorectal cancer cell HT29 proliferation was repressed. Activation of GABA_B_R could arrest the cell at G1 phase and reduce cells in S, and G2/M phases, which indicated that activation of GABA_B_R signaling could inhibit the colorectal cell HT29 proliferation by arresting the cell at G1 phase. The G1 phase‐related gene cyclin D1 was downregulated while the cell cycle inhibitor Rb1, Rbl1, and P21 were upregulated by inhibition of GABA_B_R, which helped us determined that activation of GABA_B_R can significantly promoted colorectal cancer cell proliferation by arrested cell at G1 phase.

Furthermore, to investigate how GABA_B_R regulated colorectal cancer cell proliferation and find its downstream moleculars, we considered about GSK‐3*β* and NF‐*κ*B. The phosphoinositide 3‐kinase/Akt/glycogen synthase kinase‐3*β* (GSK‐3*β*) and Wnt/*β*‐catenin pathways are downregulated in a number of cancers, and these two pathways share a common node protein, GSK‐3*β*. This protein is responsible for the regulation/degradation of *β*‐catenin, which reduces *β*‐catenin's translocation to the nucleus and influences the subsequent transcription of oncogenes 23, 25, 26. GSK‐3*β* is proved to direct proliferation of breast cancer cells interplayed with histone H3 phosphorylation and DNA methylation. Silencing of GSK‐3*β* by shRNA prevented histone H3 phosphorylation and reduced DNMT1 expression so that can restrain breast cancer cell proliferation 27. The other, NF‐*κ*B, represents an evolutionarily conserved family of inducible transcription factors that controls a large set of physiological processes ranging from basic inflammatory responses and innate and acquired immunity to the regulation of cell death such as apoptosis, autophagy, and senescence. In addition, NF‐*κ*B coordinates the expression of specific genes that mediates proliferation, cell adhesion, and differentiation 28, 29. Moreover, in several cancers, NF‐*κ*B is constitutively activated and drives tumor cell survival and proliferation 30. For many years the NF‐*κ*B signaling pathway has attracted much interest because of the possibility of targeting it for the treatment of inflammatory diseases and cancer. Therefore, interfering more upstream in the NF‐*κ*B signaling cascade is expected to be much more specific and to cause fewer side effects 31. Signaling upstream of NF‐*κ*B is quite complex and involves multiple protein–protein interactions and posttranslational modifications so there's still more that we can do and we are working on this 32.

Our study revealed that activation of GABA_B_R could increase the phosphorylation level of GSK‐3*β* which means GABA_B_R could regulate the activity of GSK‐3*β*. Furthermore, we found that inhibition of GSK‐3*β* activation, which leads to repression of colorectal cancer cell proliferation, can be rescued by upregulation of NF‐*κ*B activation by PMA. Then, we activated GABA_B_R finding that the activation of NF‐*κ*B was promoted so that the colorectal cancer cell proliferation was inhibited. The promotion of proliferation caused by downregulation of GABA_B_R can be blocked by inhibition of NF‐*κ*B activation which meant that GABA_B_R could regulate cell proliferation though regulating activation of NF‐*κ*B.

These results might provide more insight into the specific roles of GABA_B_R/GSK‐3*β*/NF‐*κ*B signaling pathway in colorectal cancer cell proliferation to lay the foundation for further clinical application.

## Conflict of Interest

None declared.

## Supporting information


**Figure S1.** related to figure 1 GABABR regulates the proliferation of HCT116 cells.

**Figure S2.** related to figure 2. GABA_B_R signaling repressed HCT116 cell proliferation by inhibiting GSK‐3β activation.

**Figure S3.** related to figure 3. GSK‐3β/NF‐κB signaling regulates the HCT116 cell proliferation.

**Figure S4.** related to figure 4. GABA_B_R/NF‐κB signaling pathway regulates the proliferation in HCT116 cells.

## References

[1] Siegel, R. , D. Naishadham , and A. Jemal . 2013 Cancer statistics, 2013. CA Cancer J. Clin. 63:11–30. 23335087 10.3322/caac.21166

[2] Jiang, X. , L. Su , Q. Zhang , C. He , Z. Zhang , P. Yi , et al. 2012 GABAB receptor complex as a potential target for tumor therapy. J. Histochem. Cytochem. 60:269–279. 22266766 10.1369/0022155412438105 PMC3351242

[3] Jemal, A. , F. Bray , M. M. Center , J. Ferlay , E. Ward , and D. Forman . 2011 Global cancer statistics. CA Cancer J. Clin. 61:69–90. 21296855 10.3322/caac.20107

[4] Lampropoulos, P. , A. Zizi‐Sermpetzoglou , S. Rizos , A. Kostakis , N. Nikiteas , and A. G. Papavassiliou . 2012 TGF‐beta signalling in colon carcinogenesis. Cancer Lett. 314:1–7. 22018778 10.1016/j.canlet.2011.09.041

[5] Vaiopoulos, A. G. , I. D. Kostakis , M. Koutsilieris , and A. G. Papavassiliou . 2012 Colorectal cancer stem cells.Stem Cells 30:363–371. 22232074 10.1002/stem.1031

[6] Wang, T. , W. Huang , and F. Chen . 2008 Baclofen, a GABAB receptor agonist, inhibits human hepatocellular carcinoma cell growth in vitro and in vivo. Life Sci. 82:536–541. 18222491 10.1016/j.lfs.2007.12.014

[7] Opolski, A. , M. Mazurkiewicz , J. Wietrzyk , Z. Kleinrok , and C. Radzikowski . 2000 The role of GABA‐ergic system in human mammary gland pathology and in growth of transplantable murine mammary cancer. J. Exp. Clin. Cancer Res. 19:383–390. 11144533

[8] Li, H. , K. Huang , X. Liu , J. Liu , X. Lu , K. Tao , et al. 2014 Lithium chloride suppresses colorectal cancer cell survival and proliferation through ROS/GSK‐3beta/NF‐kappaB signaling pathway. Oxid. Med. Cell Longev. 2014:241864. 25002914 10.1155/2014/241864 PMC4070474

[9] Luo, J. 2009 Glycogen synthase kinase 3beta (GSK3beta) in tumorigenesis and cancer chemotherapy. Cancer Lett. 273:194–200. 18606491 10.1016/j.canlet.2008.05.045 PMC4978950

[10] Cao, Q. , X. Lu , and Y. J. Feng . 2006 Glycogen synthase kinase‐3beta positively regulates the proliferation of human ovarian cancer cells. Cell Res. 16:671–677. 16788573 10.1038/sj.cr.7310078

[11] Gavilan, E. , S. Giraldez , I. Sanchez‐Aguayo , F. Romero , D. Ruano , and P. Daza . 2015 Breast cancer cell line MCF7 escapes from G1/S arrest induced by proteasome inhibition through a GSK‐3beta dependent mechanism. Sci. Rep. 5:10027. 25941117 10.1038/srep10027 PMC4419540

[12] Vaiopoulos, A. G. , K. K. Papachroni , and A. G. Papavassiliou . 2010 Colon carcinogenesis: learning from NF‐kappaB and AP‐1. Int. J. Biochem. Cell Biol. 42:1061–1065. 20348011 10.1016/j.biocel.2010.03.018

[13] Xia, Y. , S. Shen , and I. M. Verma . 2014 NF‐kappaB, an active player in human cancers. Cancer Immunol. Res. 2:823–830. 25187272 10.1158/2326-6066.CIR-14-0112 PMC4155602

[14] Smale, S. T. 2011 Hierarchies of NF‐kappaB target‐gene regulation. Nat. Immunol. 12:689–694. 21772277 10.1038/ni.2070 PMC3169328

[15] Wang, Y. , S. Baskerville , A. Shenoy , J. E. Babiarz , L. Baehner , and R. Blelloch . 2008 Embryonic stem cell‐specific microRNAs regulate the G1‐S transition and promote rapid proliferation. Nat. Genet. 40:1478–1483. 18978791 10.1038/ng.250 PMC2630798

[16] Huang, Q. , C. L. Zhu , C. H. Liu , F. Xie , K. Zhu , and S. Y. Hu . 2013 Gamma‐aminobutyric acid binds to GABAb receptor to inhibit cholangiocarcinoma cells growth via the JAK/STAT3 pathway. Dig. Dis. Sci. 58:734–743. 23007731 10.1007/s10620-012-2382-2

[17] Maemura, K. , N. Shiraishi , K. Sakagami , K. Kawakami , T. Inoue , M. Murano , et al. 2009 Proliferative effects of gamma‐aminobutyric acid on the gastric cancer cell line are associated with extracellular signal‐regulated kinase 1/2 activation. J. Gastroenterol. Hepatol. 24:688–696. 19032445 10.1111/j.1440-1746.2008.05687.x

[18] Liu, Y. , F. Guo , M. Dai , D. Wang , Y. Tong , J. Huang , et al. 2009 Gammaaminobutyric acid A receptor alpha 3 subunit is overexpressed in lung cancer. Pathol. Oncol. Res. 15:351–358. 19048400 10.1007/s12253-008-9128-7

[19] Minuk, G. Y. , M. Zhang , Y. Gong , L. Minuk , H. Dienes , N. Pettigrew , et al. 2007 Decreased hepatocyte membrane potential differences and GABAA‐beta3 expression in human hepatocellular carcinoma. Hepatology 45:735–745. 17326191 10.1002/hep.21562

[20] Fava, G. , L. Marucci , S. Glaser , H. Francis , S. De Morrow , A. Benedetti , et al. 2005 Gamma‐Aminobutyric acid inhibits cholangiocarcinoma growth by cyclic AMP‐dependent regulation of the protein kinase A/extracellular signal‐regulated kinase 1/2 pathway. Cancer Res. 65:11437–11446. 16357152 10.1158/0008-5472.CAN-05-1470

[21] Watanabe, M. , K. Maemura , K. Kanbara , T. Tamayama , and H. Hayasaki . 2002 GABA and GABA receptors in the central nervous system and other organs. Int. Rev. Cytol. 213:1–47. 11837891 10.1016/s0074-7696(02)13011-7

[22] Thaker, P. H. , K. Yokoi , N. B. Jennings , Y. Li , R. B. Rebhun , D. L. Jr Rousseau , et al. 2005 Inhibition of experimental colon cancer metastasis by the GABA‐receptor agonist nembutal. Cancer Biol. Ther. 4:753–758. 15970706 10.4161/cbt.4.7.1827

[23] Grimes, C. A. , and R. S. Jope . 2001 The multifaceted roles of glycogen synthase kinase 3beta in cellular signaling. Prog. Neurobiol. 65:391–426. 11527574 10.1016/s0301-0082(01)00011-9

[24] Zhang, D. , X. Li , Z. Yao , C. Wei , N. Ning , and J. Li . 2014 GABAergic signaling facilitates breast cancer metastasis by promoting ERK1/2‐dependent phosphorylation. Cancer Lett. 348:100–108. 24657659 10.1016/j.canlet.2014.03.006

[25] Atkins, R. J. , J. Dimou , L. Paradiso , A. P. Morokoff , A. H. Kaye , K. J. Drummond , et al. 2012 Regulation of glycogen synthase kinase‐3 beta (GSK‐3beta) by the Akt pathway in gliomas. J. Clin. Neurosci. 19:1558–1563. 22999562 10.1016/j.jocn.2012.07.002

[26] Jope, R. S. 2003 Lithium and GSK‐3: one inhibitor, two inhibitory actions, multiple outcomes. Trends Pharmacol. Sci. 24:441–443. 12967765 10.1016/S0165-6147(03)00206-2

[27] Gupta, C. , J. Kaur , and K. Tikoo . 2014 Regulation of MDA‐MB‐231 cell proliferation by GSK‐3beta involves epigenetic modifications under high glucose conditions. Exp. Cell Res. 324:75–83. 24704462 10.1016/j.yexcr.2014.03.019

[28] Karin, M. , and Y. Ben‐Neriah . 2000 Phosphorylation meets ubiquitination: the control of NF‐[kappa]B activity. Annu. Rev. Immunol. 18:621–663. 10837071 10.1146/annurev.immunol.18.1.621

[29] Hayden, M. S. , and S. Ghosh . 2008 Shared principles in NF‐kappaB signaling. Cell 132:344–362. 18267068 10.1016/j.cell.2008.01.020

[30] DiDonato, J. A. , F. Mercurio , and M. Karin . 2012 NF‐kappaB and the link between inflammation and cancer. Immunol. Rev. 246:379–400. 22435567 10.1111/j.1600-065X.2012.01099.x

[31] Hinz, M. , and C. Scheidereit . 2014 The IkappaB kinase complex in NF‐kappaB regulation and beyond. EMBO Rep. 15:46–61. 24375677 10.1002/embr.201337983 PMC4303448

[32] Verstrepen, L. , and R. Beyaert . 2014 Receptor proximal kinases in NF‐kappaB signaling as potential therapeutic targets in cancer and inflammation. Biochem. Pharmacol. 92:519–529. 25449604 10.1016/j.bcp.2014.10.017

