# Supplementary figures and images for: GABABR/GSK‐3β/NF‐κB signaling pathway regulates the proliferation of colorectal cancer cells
Source: Cancer Med. 2016 Apr 5;5(6):1259–67. doi: 10.1002/cam4.686 (PMC4924384; doi:10.1002/cam4.686)

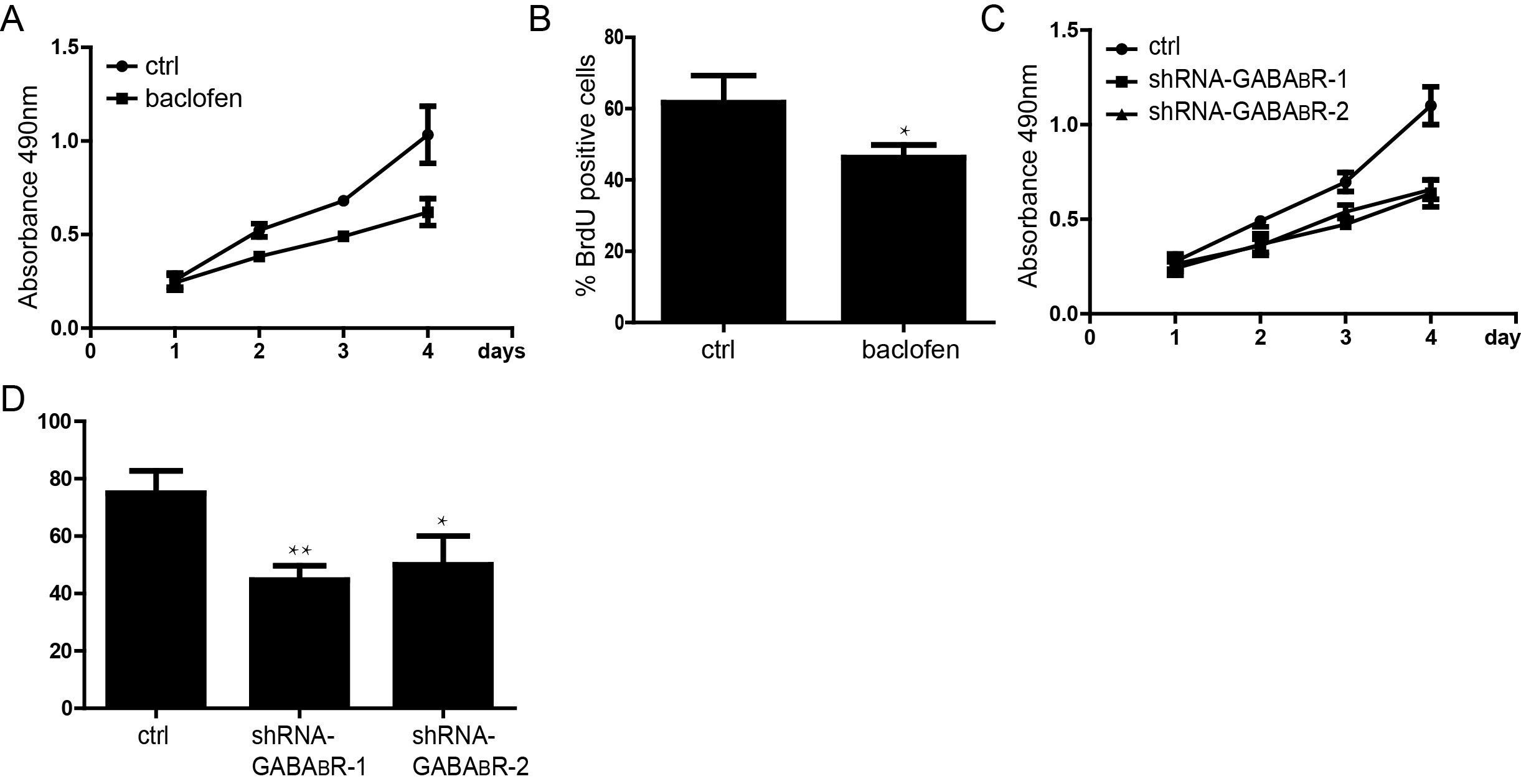

Supplement: Supplementary file 1 — Figure S1. related to figure 1 GABABR regulates the proliferation of HCT116 cells. [file CAM4-5-1259-s001.tif]

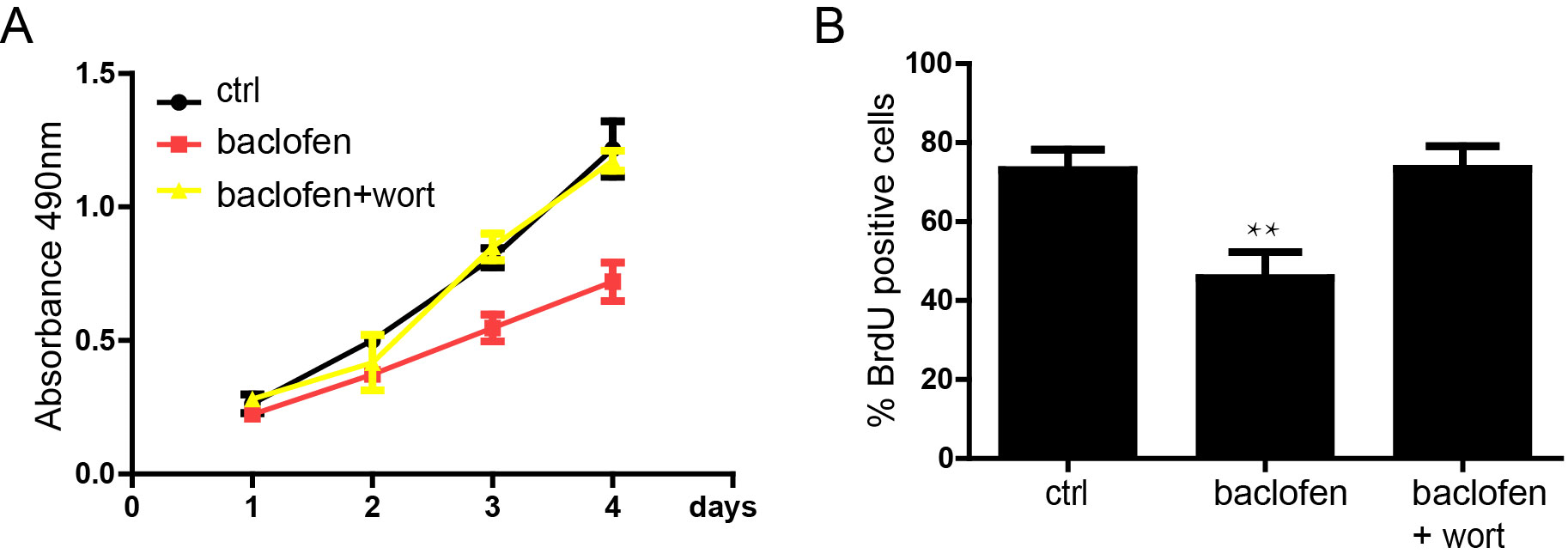

Supplement: Supplementary file 2 — Figure S2. related to figure 2. GABABR signaling repressed HCT116 cell proliferation by inhibiting GSK‐3β activation. [file CAM4-5-1259-s002.tif]

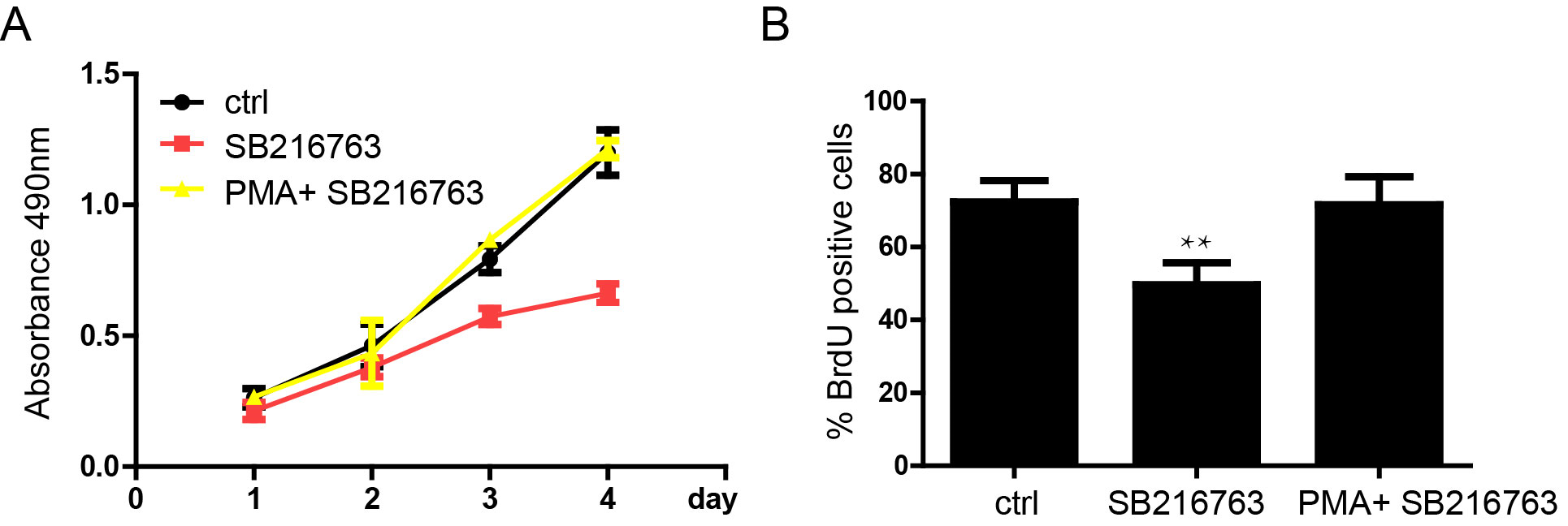

Supplement: Supplementary file 3 — Figure S3. related to figure 3. GSK‐3β/NF‐κB signaling regulates the HCT116 cell proliferation. [file CAM4-5-1259-s003.tif]

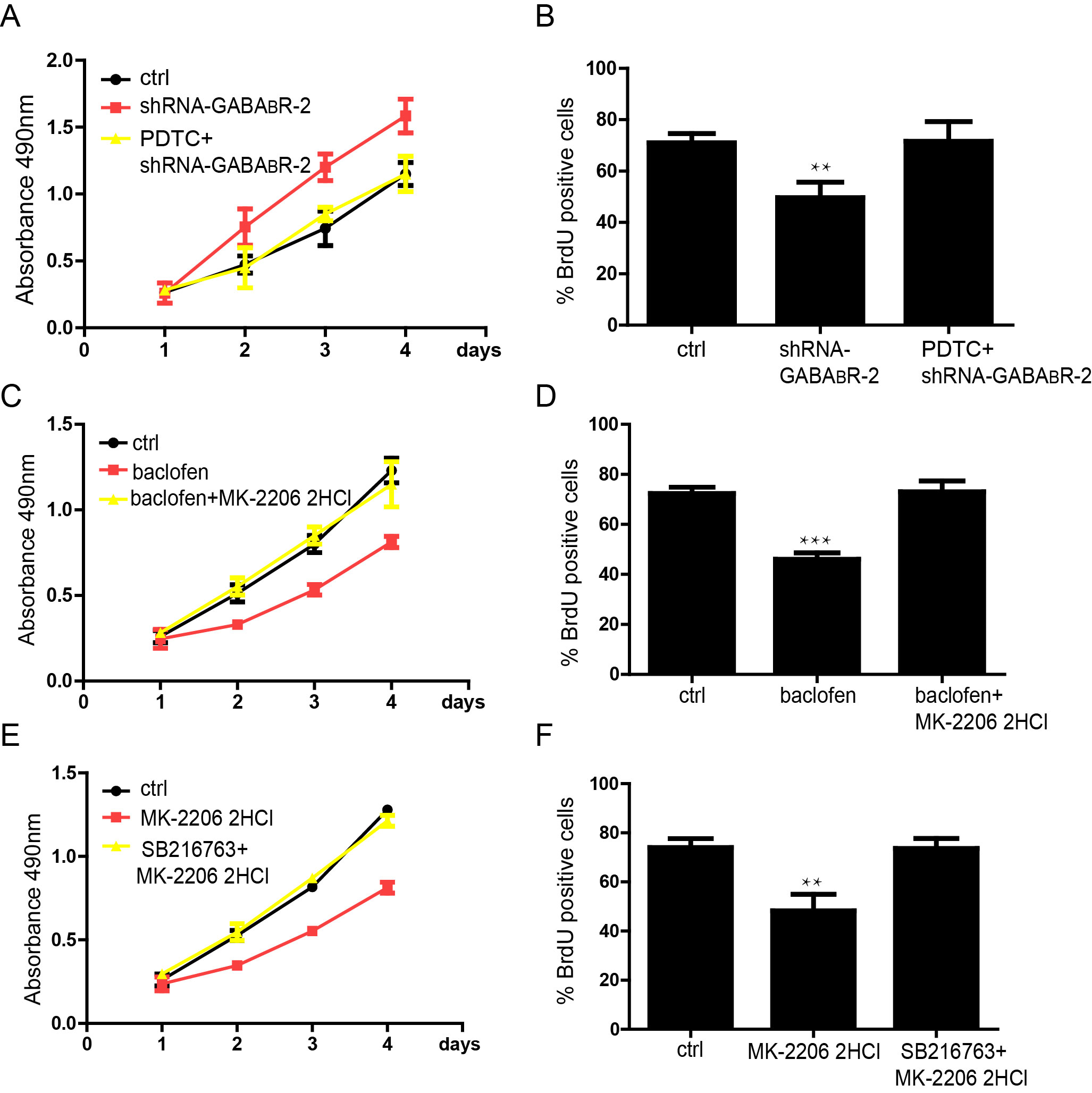

Supplement: Supplementary file 4 — Figure S4. related to figure 4. GABABR/NF‐κB signaling pathway regulates the proliferation in HCT116 cells. [file CAM4-5-1259-s004.tif]
